# Genome-wide analysis of the KNOX gene family in Moso bamboo: insights into their role in promoting the rapid shoot growth

**DOI:** 10.1186/s12870-024-04883-2

**Published:** 2024-03-25

**Authors:** Yang Jiao, Jiaqi Tan, Hui Guo, Bin Huang, Yeqing Ying, Muthusamy Ramakrishnan, Zhijun Zhang

**Affiliations:** 1 Bamboo Industry Institute, Zhejiang A&F University, Lin’an, Hangzhou, 311300 Zhejiang China; 2 State Key Laboratory of Tree Genetics and Breeding, Co-Innovation Center for Sustainable Forestry in Southern China, Bamboo Research Institute, Key Laboratory of National Forestry and Grassland Administration on Subtropical Forest Biodiversity Conservation, School of Life Sciences, Nanjing Forestry University, Nanjing, 210037 Jiangsu China

**Keywords:** KNOX gene family, Moso bamboo, Rapid growth, *Cis*-acting elements, Gene expression, Evolutionary, Downstream target genes

## Abstract

**Background:**

*KNOTTED1*-like homeobox (*KNOX*) genes, plant-specific homologous box transcription factors (TFs), play a central role in regulating plant growth, development, organ formation, and response to biotic and abiotic stresses. However, a comprehensive genome-wide identification of the *KNOX* genes in Moso bamboo (*Phyllostachys edulis*), the fastest growing plant, has not yet been conducted, and the specific biological functions of this family remain unknown.

**Results:**

The expression profiles of 24 *KNOX* genes, divided into two subfamilies, were determined by integrating Moso bamboo genome and its transcriptional data. The *KNOX* gene promoters were found to contain several light and stress-related *cis*-acting elements. Synteny analysis revealed stronger similarity with rice *KNOX* genes than with *Arabidopsis KNOX* genes. Additionally, several conserved structural domains and motifs were identified in the KNOX proteins. The expansion of the KNOX gene family was primarily regulated by tandem duplications. Furthermore, the *KNOX* genes were responsive to naphthaleneacetic acid (NAA) and gibberellin (GA) hormones, exhibiting distinct temporal expression patterns in four different organs of Moso bamboo. Short Time-series Expression Miner (STEM) analysis and quantitative real-time PCR (qRT-PCR) assays demonstrated that *PeKNOX* genes may play a role in promoting rapid shoot growth. Additionally, Gene Ontology (GO) and Protein–Protein Interaction (PPI) network enrichment analyses revealed several functional annotations for *PeKNOXs*. By regulating downstream target genes, *PeKNOXs* are involved in the synthesis of AUX /IAA, ultimately affecting cell division and elongation.

**Conclusions:**

In the present study, we identified and characterized a total of 24 *KNOX* genes in Moso bamboo and investigated their physiological properties and conserved structural domains. To understand their functional roles, we conducted an analysis of gene expression profiles using STEM and RNA-seq data. This analysis successfully revealed regulatory networks of the *KNOX* genes, involving both upstream and downstream genes. Furthermore, the *KNOX* genes are involved in the AUX/IAA metabolic pathway, which accelerates shoot growth by influencing downstream target genes. These results provide a theoretical foundation for studying the molecular mechanisms underlying the rapid growth and establish the groundwork for future research into the functions and transcriptional regulatory networks of the KNOX gene family.

**Supplementary Information:**

The online version contains supplementary material available at 10.1186/s12870-024-04883-2.

## Background

Moso bamboo (*Phyllostachys edulis*) is the most widespread and economically/ecologically significant bamboo species in China [1]. It is a monocarpic, scattered, evergreen, tree-like bamboo species and the fastest growing plant, reaching a height of 20 m in about 60 days [2, 3]. Moso bamboo, with its abundance of lignocellulose, is both lightweight and elastic. These properties make it a favorable alternative to wood in various industries, including furniture and paper manufacturing. Its rapid growth and versatility make it a sustainable choice for many applications, potentially helping to reduce the need for excessive deforestation [1]. However, the transition mechanism that regulates its rapid growth remains unknown, despite the developments in the rapid growth [2].

Genes encoding homologous proteins in eukaryotes are divided into two classes: TALEs (three amino acid length extensions) and non-TALEs. *KNOTTED1*-like homeobox (*KNOX*) genes, belonging to the TALE superclass, regulate stem cell development in plants [4]. *KNOX* genes encode transcription factors (TFs) that specifically regulate stem-cell specification at the shoot apical meristem (SAM) [5]. KNOX genes feature an additional three amino acids (P-Y-P), setting them apart from other homozygous heterotypic cassette proteins [6]. KNOX proteins consist of several domains, including the MEINOX domain with KNOX1 and KNOX2 at the N-terminal region, an ELK domain, and the TALE-type Homeobox_KN domain at the C-terminal region [7, 8].

The KNOX gene family is divided into three classes: Class I, Class II, and Class KNATM based on phylogenetic analysis, homologous structural domain similarities, introns, and expression patterns [9]. The KNOX1 and KNOX2 domains participate in protein–protein interactions [10, 11]. The Class I and Class II of *KNOX* genes are present in bryophytes and all flowering plants and play opposite but conserved roles. In angiosperms, the Class I subfamily genes are expressed primarily in the apical meristem and play essential roles in the growth and maintenance of meristematic tissue [12, 13]. The Class I genes regulate plant organ development and secondary cell wall biosynthesis. They are also expressed in various plant organs, including roots, stems, leaves, and flowers [14].

*KNOX* genes have been studied in various plants, including gymnosperms like *Pinus sylvestris*, monocots such as rice and orchids, and dicots like *Arabidopsis*, poplars and pears [15]. In *Arabidopsis*, the Class I subfamily is exclusively at the shoot apical meristem (SAM) and includes genes such as shoot meristemless (STM), KNAT1, KNAT2, and KNAT6. For instance, STM has been identified as a positive regulator of related *KNOX* genes. The Class I genes play a vital role in maintaining floral meristem and carpel formation, influencing floral architecture [16, 17]. In contrast, the Class II gene functions, such as *KNAT3*, *KNAT4*, *KNAT5*, and *KNAT7*, remain less understood, and the Class II genes exhibit broader expression patterns in differentiating and mature tissues [18]. Genetic analyses indicate that the Class II genes promote the differentiation of all aerial organs while suppressing hyphal tissues, often acting in opposition to the Class I genes. Nevertheless, their precise functions have yet to be elucidated due to limited research and the presence of high genetic redundancy [19]. In certain dicotyledonous plants, the KNATM gene, a KNOX gene found in *Arabidopsis*, is present and lacks the Homeobox_KN structural domain. KNATM is expressed at the boundary of mature organs and in the proximal–distal domains of organ primordia [20, 21].

TFs play a critical role in actively regulating or repressing the transcription of downstream genes [22]. To understand the function of *KNOX* genes in transcription, it is imperative to identify their downstream targets. However, only a limited number of studies have delved into the downstream targets of *KNOX* genes. *KNOX* is a TF and has been shown to modulate hormone levels, particularly cytokinin and auxin [23–25], to inhibit gibberellin synthesis [26], and to participate in secondary cell wall formation [27]. Consequently, *KNOX* genes emerge as pivotal contributors to plant growth and development [28]. For example, in poplar, the KNOX gene *PagKNAT2/6b* can directly inhibit gibberellin (GA) synthesis, which inhibits cell elongation and expansion, leading to a reduction in internode length and leaf size [29]. Similarly, in tomato seedlings, the overexpression of the *KNOX* gene have increased sensitivity to growth hormone and up-regulated expression of genes involved in the control of IAA synthesis and transport [30].

Therefore, in the present study, we conducted a genome-wide identification and evolutionary analysis of the KNOX gene family in Moso bamboo. In addition, the *KNOX* downstream target genes were systematically identified. Furthermore, the expression profiles of the KNOX gene families in different shoot heights and tissue sites of Moso bamboo were analyzed using RNA-seq and quantitative real-time PCR analysis (qRT-PCR). The aim of this study was to investigate the role of *KNOX* genes in facilitating the growth and development of Moso bamboo.

## Results

### Identification and characterization of KNOX genes

Based on the HMM model of the KNOX structural domain, 49 potential *KNOX* genes were initially identified in the Moso bamboo genomic database. However, after rigorous screening, which included excluding genes with an E-value ≤ 10^-15^, only 24 KNOX family genes were remained identified. These genes were renamed *PeKNOX01*-*PeKNOX24* based on their positions on the chromosomal scaffold (Table S1). Our predictions suggested that these genes encode proteins with amino acid lengths ranging from 289 to 380, molecular weights ranging from 30 to 42 kDa, and isoelectric points ranging from 5.36 to 8.1. The thermal stability of the KNOX proteins, as measured by the aliphatic amino acid index, ranged from 62.96 to 78.66, indicating that they were all unstable proteins. In addition, all KNOX proteins had negative hydrophilicity scores (GRAVY), indicating they were all hydrophilic proteins. The subcellular localization predictions conducted using the WoLF PSORT online tool revealed that all KNOX proteins were located in the nucleus.

### KNOX gene structure and analysis of cis-acting elements

**Fig. 1:**
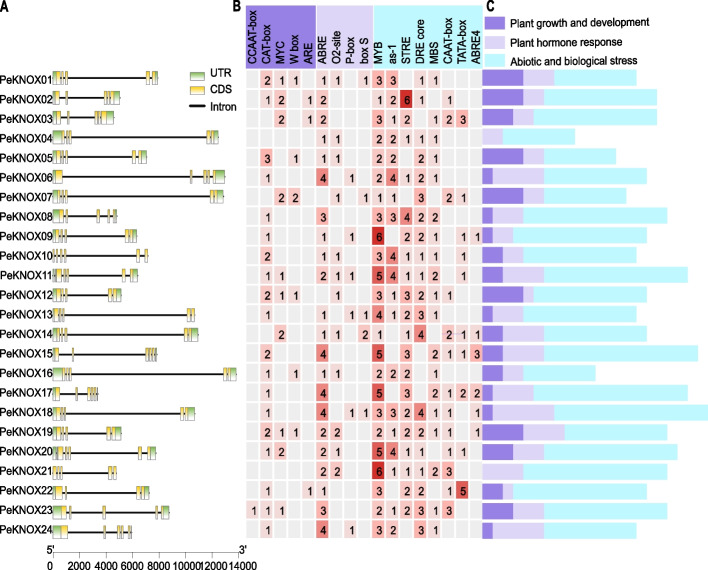
Analysis of intron–exon structures and *cis*-acting elements of *PeKNOX* gene family. **A** Intron–exon distribution of *PeKNOX* genes. **B** The histogram of the *cis*-acting elements in each gene. **C** Three most-frequent categories of *cis*-acting elements in *PeKNOXs*

The amino acid sequence characteristics of the 24 *PeKNOX* members were further analyzed by predicting conserved motifs (Table S2). The number of introns in each *KNOX* gene varied from two to five. Furthermore, the KNOX gene family contained four to six exons (Fig. 1A). Interestingly, we observed significant differences in the gene structure within the KNOX gene families. For instance, *PeKNOX10* and *PeKNOX21* lacked non-coding regions, while *PeKNOX13* contained one non-coding region. All other genes contained two non-coding regions, which might be attributed to the absence of annotation information in the corresponding untranslated region (UTR) section of the GFF file. Using the PlantCARE software, we also identified *cis*-acting elements located 1500 base pairs upstream of *PeKNOX* gene. Among the hormone response-related *cis*-regulatory components, ABRE was the most common element associated with abscisic acid reaction (Fig. 1B). These *cis*-acting elements were categorized into three groups based on their associated functions, including plant growth and development, plant hormone response, and biotic and abiotic stresses (Fig. 1C).

### Phylogenetic tree and conserved motifs and domains

**Fig. 2:**
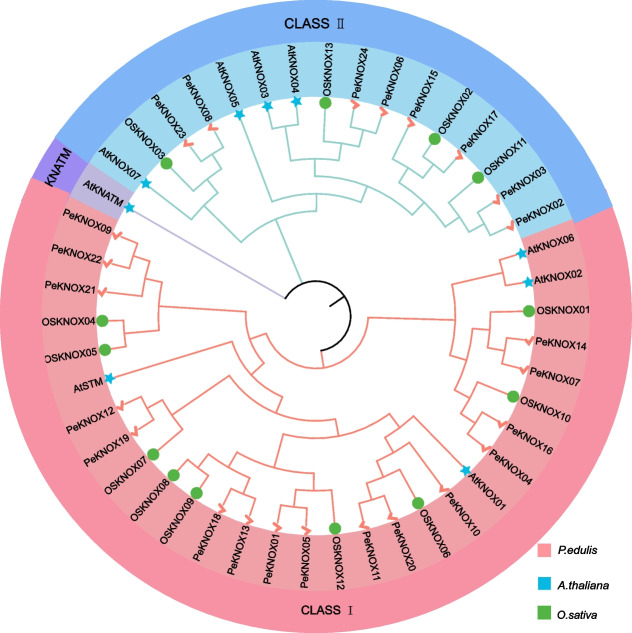
Phylogenetic analysis of KNOX proteins extracted from *P. edulis*, *Arabidopsis*, and *O. sativa*. In the phylogenetic tree, pink checkmarks indicate *P. edulis*, blue stars indicate *Arabidopsis*, and green circles indicate *O. sativa*. The tree was constructed based on the Neighbor-joining algorithm using MEGA 7, with 1000 bootstrap replicates

Homologous genes tend to have similar structures and functions. To study the evolutionary relationship between *Arabidopsis* and the corresponding *KNOX* genes in rice and Moso bamboo, a phylogenetic tree was constructed using the Neighbor-Joining method. This analysis involved the amino acid sequences of 24 Moso bamboo *KNOX* genes, 9 *Arabidopsis KNOX* genes, and 13 rice *KNOX* genes. The phylogenetic tree revealed that all KNOX family members were divided into three subcategories: Class I, Class II, and KNATM. In Class I, there were 29 members—comprising Moso bamboo (16), rice (9), and *Arabidopsis* (4). Class II comprised 16 members: 8 from Moso bamboo, 4 from rice, and 4 from *Arabidopsis*, while the KNATM class featured a single gene from *Arabidopsis*. Notably, *Arabidopsis*, as a dicotyledonous plant, exhibited a distinct evolutionary relationship compared to Moso bamboo and rice. This divergence might be attributed to the potential gene loss and degradation that dicotyledons can undergo during evolution. Consequently, the phylogenetic analysis underscored that the homology of KNOX proteins with *Arabidopsis* was considerably lower than their similarity to rice KNOX proteins (Fig. 2).

**Fig. 3:**
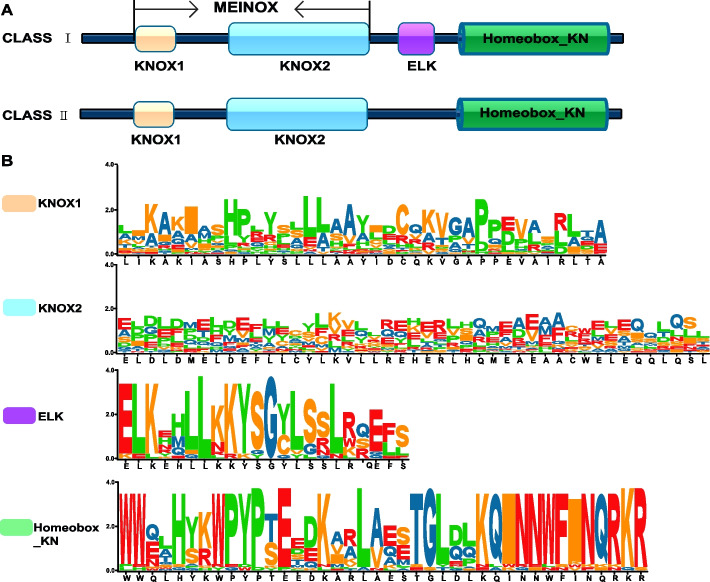
The domain compositions of PeKNOX proteins. **A** Schematic illustration of the four domain structures. **B** Amino acids’ sequence logos for the KNOX1, KNOX2, ELK, and Homeobox_KN domain

Multiple sequence alignments were conducted to determine the location and characteristics of the conserved protein domains in Moso bamboo. The alignments revealed that all KNOX family members contained highly conserved KNOX1, KNOX2, and Homeobox_KN regions (Fig. 3A), each consisting of approximately 40–50 amino acids (Fig. 3B). Furthermore, Class I family members had a highly conserved ELK region, composed of about 30 amino acids (Fig. 3A).

**Fig. 4:**
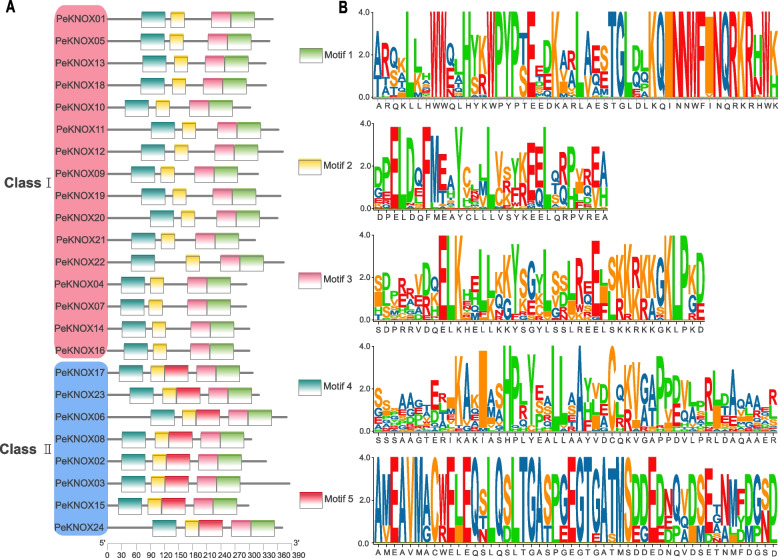
Conserved motifs of the KNOX proteins identified using the MEME/MAST system. **A** Distribution of five conserved motifs. **B** The corresponding legends and logos for the five motifs as visualized for PeKNOXs

Furthermore, the analysis of the *KNOX* gene structure in Moso bamboo and motif composition revealed five highly conserved motifs (Fig. 4A). The motif logo is shown on the right in Fig. 4B. The stable structure consisted of five motifs (motif 1, 2, 3, 4, and 5). The relative positions of these motifs were consistent across most sequences. Class I family members lacked motif 5, possibly due to a loss of the KNOX family over the evolutionary process.

### Chromosomal location and gene duplication of PeKNOX genes

**Fig. 5:**
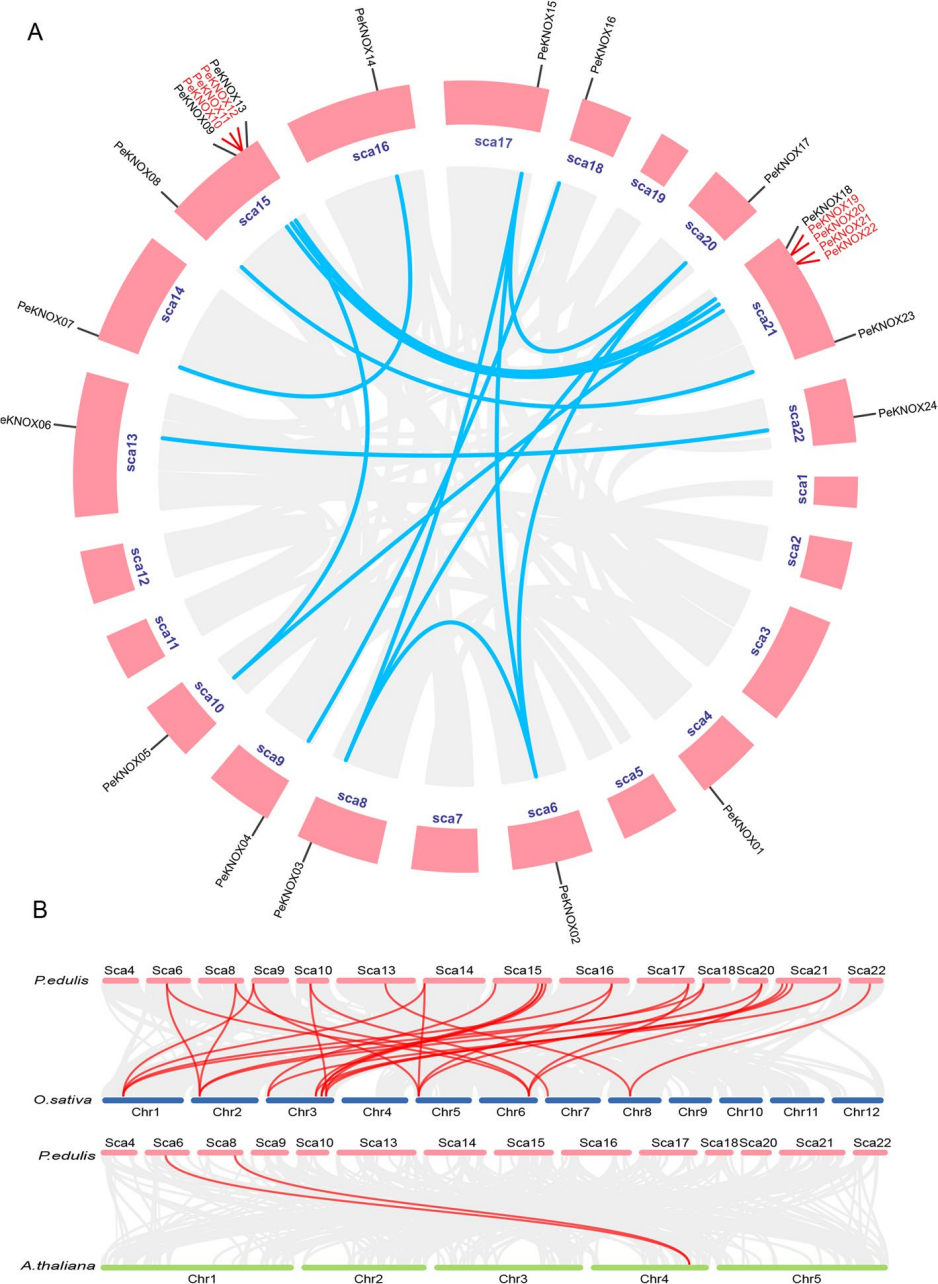
Syntenic analysis of *KNOX* genes. **A** Chromosomal distribution and interchromosomal relationships among *PeKNOX* genes. **B** Interspecies collinearity analysis of *KNOX* genes among *P. edulis*, *Arabidopsis*, and *O. sativa*

TBtools was used to map the 24 *KNOX* genes to their respective chromosome locations in Moso bamboo (Fig. 5A). Most genes were located on Sca15 (6) and Sca22 (6), while only one gene was identified on each of the remaining 12 chromosomes (Sca4, Sca6, Sca8, Sca9, Sca10, Sca13, Sca14, Sca16, Sca17, Sca18, Sca20, and Sca22). Furthermore, we observed small-scale gene clusters on chromosomes 15 and 22, probably due to genetic separation resulting from crossover and recombination events. Gene duplication is common among all species and leads to the evolution of novel and beneficial genes. To investigate duplications in the KNOX gene family in Moso bamboo, MCScanX genome synteny analysis was employed to identify 16 gene pairs resulting from segmental duplications. The results revealed that *PeKNOX01* and *PeKNOX12* were not involved in gene duplications. Typically, closely related genes are found in close proximity, often due to tandem duplication events. To further investigate gene duplications in the KNOX gene family, genome-to-genome homologous analysis was conducted between Moso bamboo and *Arabidopsis* and rice (Fig. 5B). The results revealed that *PeKNOX02* and *PeKNOX03* were syntenic with genes from rice and *Arabidopsis*. Moreover, Moso bamboo possesses orthologs for each rice KNOX gene, many of which have multiple orthologs, suggesting that additional genome-wide duplication events may have occurred during Moso bamboo evolution.

### Analysis of transcriptional expression patterns

**Fig. 6:**
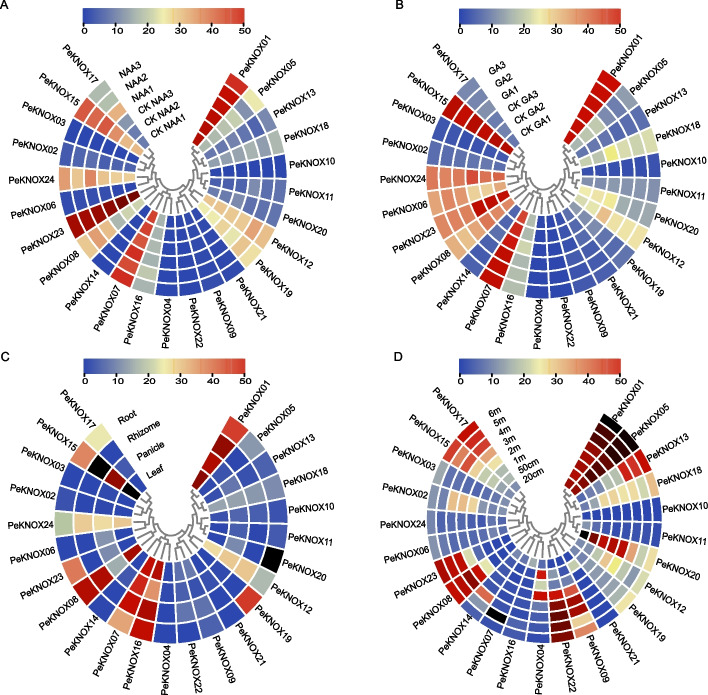
Heatmaps showing the expression patterns of *PeKNOX* genes based on the transcriptome datasets of Moso bamboo (PRJEB2956, PRJNA414226, and PRJNA413166). Analysis of the expression patterns of NAA treatment (**A**), GA treatment (**B**), different tissues/organs such as roots, rhizomes, panicle, and leaves (**C**), and different heights of shoots such as 20 cm, 50 cm, 1 m, 2 m, 3 m, 4 m, 5 m, and 6 m (**D**). The color scale indicates transcript abundance, with red representing high levels and blue indicating low transcript abundance

To assess the transcriptional expression patterns of *PeKNOX* genes in various tissues of Moso bamboo, including roots, rhizomes, panicle, leaves, and early shoots of different heights, we utilized 31 distinct transcriptome datasets (PRJEB2956, PRJNA414226, and PRJNA413166) of Moso bamboo downloaded from the Short Read Archive (SRA) available on the NCBI database. Additionally, we analyzed transcriptomic data obtained from the seedling treated with naphthaleneacetic acid (NAA) and gibberellin (GA) to investigate *PeKNOX* gene expression patterns (PRJNA413166). After normalization, a heat map for differential gene expression was constructed (Fig. 6). Notably, *PeKNOX* genes were highly expressed following treatment with NAA compared with the controls (Fig. 6A), indicating that NAA promotes the expression of *PeKNOX* genes. However, a marked reduction in *PeKNOX* gene expression was observed following GA treatment (Fig. 6B).

Furthermore, we observed tissue-specific expression patterns among different Moso bamboo organs (Fig. 6C). *PeKNOX01*, *PeKNOX07, PeKNOX08*, and *PeKNOX16–PeKNOX20* were highly expressed in bamboo roots, suggesting that these genes play a significant role in promoting root growth. Further, different Moso bamboo organs showed differential expression of *PeKNOX* (Fig. 6C). *PeKNOX01, PKNOX07,* and *PKNOX15* were highly expressed in panicles, whereas *PKNOX06, PKNOX07, PKNOX15*, and *PKNOX16* were highly expressed in leaves. In contrast, *PeKNOX20* exhibited high expression in the roots. Moreover, *PeKNOX01*, *PeKNOX05*, and *PeKNOX20* showed differential expression in bamboo shoots at different rapid shoot growth phases, as shown in Fig. 6D. The results revealed that *PeKNOX01*, *PeKNOX05*, and *PeKNOX20* were highly expressed in 20-cm bamboo shoots and moderately expressed in 50-cm bamboo shoots. The expression of these *PeKNOX* genes was only observed during the rapid shoot growth phase, suggesting their importance during this period.

### GO enrichment analysis and protein interaction networks

**Fig. 7:**
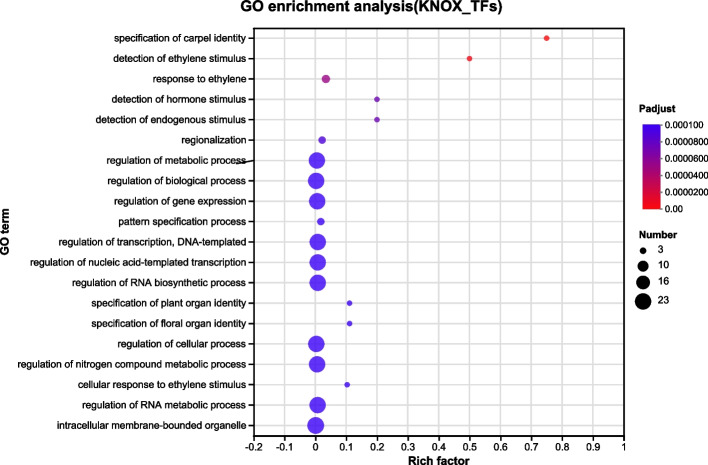
Gene Ontology (GO) annotations of the *KNOX* genes. The size of the dots indicates the number of genes in the GO term, and the color of the dots corresponds to the range of *P*-values. Enrichment results are displayed for *P*-value < 0.05

PeKNOX proteins were analyzed for GO enrichment, and a protein–protein interaction network was constructed to identify the biological processes, molecular functions, and cellular components associated with this gene family. The GO analysis revealed that 80% of *PeKNOX* genes were involved in various biological processes related to the nitrogen compounds of DNA templates, such as RNA metabolism and biosynthesis, nucleic acid transcription regulation, and transcription regulation (Fig. 7). Furthermore, *PeKNOX* proteins possess transcription activation activity and bind to specific DNA sequences to carry out their functions.

**Fig. 8:**
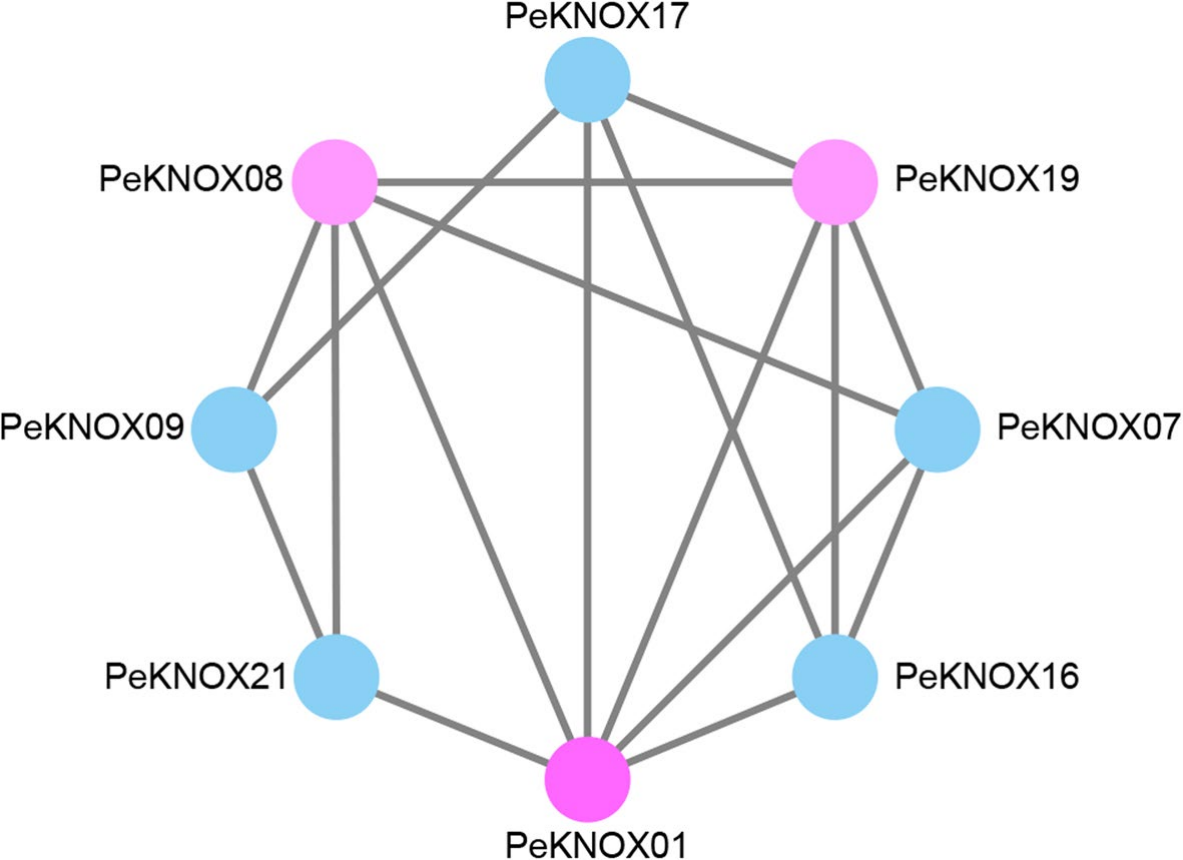
Protein–protein interaction analysis of PeKNOX proteins. Nodes indicate proteins, and edges indicate the presence of interactions between two proteins

The STRING database was used to predict potential interactions between PeKNOX proteins. Subsequently, Cytoscape was used for the visualization of the PPI networks (Fig. 8). The results revealed that some proteins exhibit complex polygenic interactions, as seen with PeKNOX01, PeKNOX08, and PeKNOX19. In addition, there were at least three connecting lines between each node, suggesting that PeKNOX proteins could be involved in transcriptional regulation through interactions among its various members.

### Expression patterns of the KNOX genes during the rapid shoot growth

**Fig. 9:**
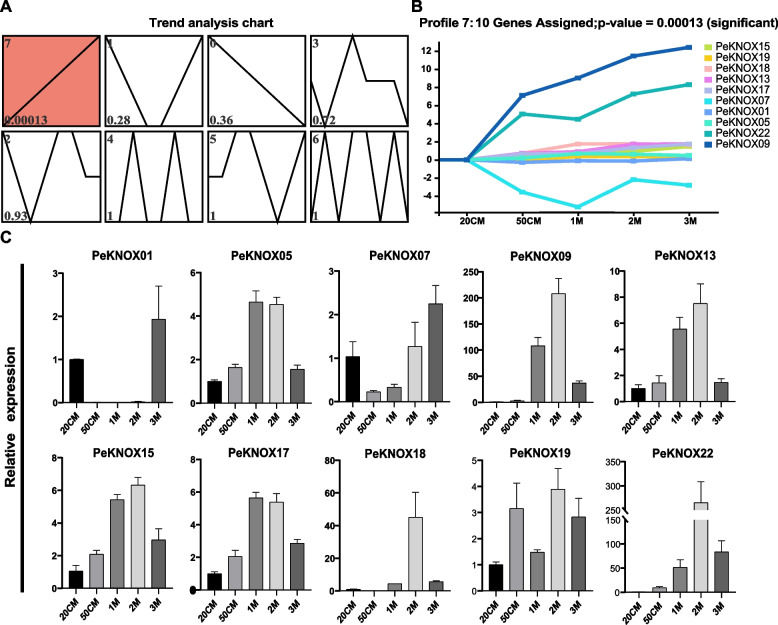
STEM analysis of the *KNOX* genes. **A** Temporal trend analysis of the *KNOX* genes. The red boxes indicate significantly expressed genes. All 8 profiles are drawn with profile numbers denoted at the top left. The value in the lower-left corner is the P-value for its corresponding significance level. **B** The characteristics of significantly expressed *PeKNOX* genes in profile 7. **C** Quantitative real-time PCR analysis (qRT-PCR) of the selected *PeKNOX* genes in profile 7. Different heights of Moso bamboo shoots are represented by 20 cm, 50 cm, 1 m, 2 m, and 3 m

Analysis of *PeKNOX* gene expression profiles in Moso bamboo shoots at different heights revealed eight distinct expression profiles (Fig. 9). Profile 7 showed a positive correlation with shoot development, suggesting that the genes in this profile could be involved in shoot growth and development (Fig. 9B). The analysis of qRT-PCR of the selected *PeKNOX* genes from profile 7 was conducted to validate the expression of genes within profile 7, and the analysis (Fig. 9C) revealed that expression patterns of genes in profile 7 are highly positively associated with shoot growth stages.

### Subcellular localization

**Fig. 10:**
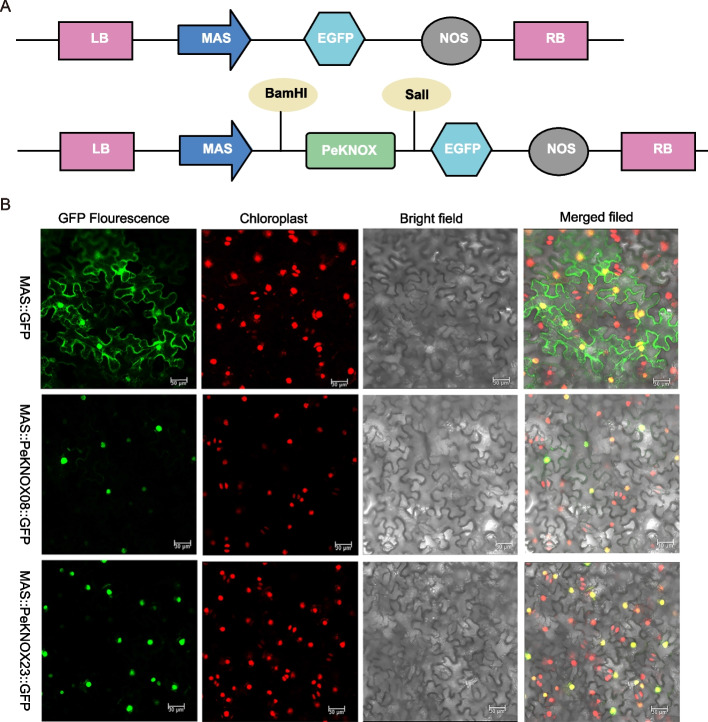
Subcellular localization of the GFP-fused PeKNOX protein. **A** The schematic representation of the vector construction for the subcellular localization of PeKNOX. **B** The subcellular localization of the fusion proteins of MAS::*PeKNOX08*::GFP, MAS::*PeKNOX23*::GFP, and control vector. Observation of GFP fluorescence of transiently transformed tobacco leaves by confocal laser scanning microscopy. Scale bar = 50 μm

The fusion proteins MAS::PeKNOX08::GFP and MAS::PeKNOX23::GFP were established in vivo through the infection of tobacco with *Agrobacterium tumefaciens* strain GV3101 (Fig. 10A). The fluorescent signal of the fusion protein was distinct from that of the control MAS::GFP and could only be seen in the nucleus. These results demonstrate that PeKNOX protein is mainly present in the nucleus (Fig. 10B).

### Construction of the KNOX gene regulatory networks and GO analysis

**Fig. 11:**
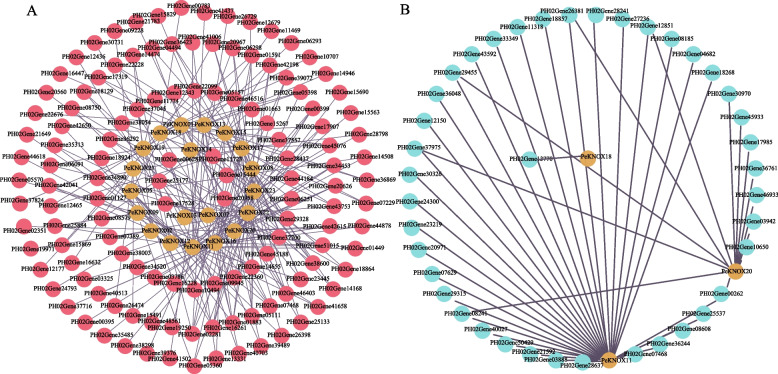
*PeKNOX* gene regulatory networks in Moso bamboo. **A** Predicted network diagram of upstream transcription factors of *PeKNOX* genes. **B** Predictive network of downstream target genes regulated by *PeKNOX* transcription factors. Blue color represents *PeKNOX* genes, and orange color represents upstream transcription factors, while downstream target genes are represented as purple nodes

**Fig. 12:**
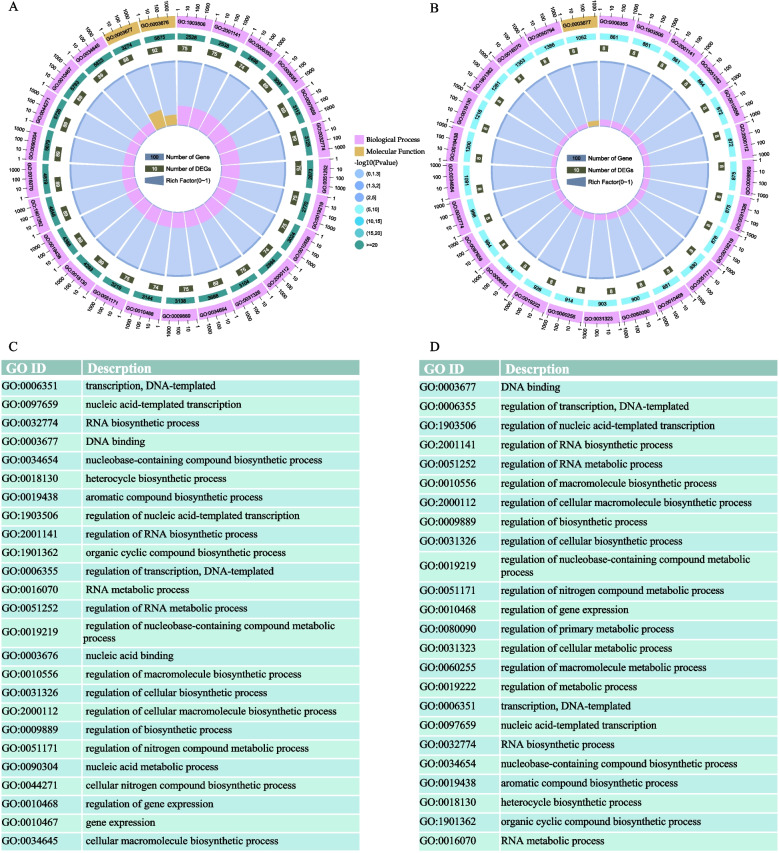
GO enrichment analysis of *PeKNOX* gene regulatory network in Moso bamboo. **A** GO enrichment analysis of upstream transcription factors of *PeKNOX* genes. **B** GO enrichment analysis of *PeKNOX* target genes. **C** The functions and GO enrichment terms of the upstream transcription factors of *PeKNOX* genes. **D** The functions and GO enrichment terms of *PeKNOX* target genes

To determine the role of *KNOX* genes in Moso bamboo, we constructed gene regulatory networks with *KNOX* genes as the central focus. The predicted regulatory network played a key role in identifying new regulatory links among genes. The analysis revealed that a total of 127 TFs regulate 20 *PeKNOX* genes (Fig. 11A). Of these, several genes, such as *PeKNOX11*, *KNOX18*, and *PeKNOX20*, function as TFs and regulate 45 corresponding downstream target genes (Fig. 11B). The GO enrichment analysis was performed for the corresponding target genes, and the top 25 enriched GO terms were shown in descending order of *P*-value. The outer ring displays the top 25 enriched GO term IDs categorized by colors. The second ring shows the number of background genes and the *P*-value indicating the significant enrichment. The third ring represents the number of *PeKNOX* genes annotated with each term. The fourth ring indicates the enrichment factor, moving from outer to center (Fig. 12A, B). GO function enrichment indicated that they were mainly enriched in molecular functions and biological processes. Upstream TFs are significantly enriched in nucleic acid binding (GO:0003676) and DNA-templated transcription (GO:0006351) (Fig. 12C). Downstream target genes are enriched in DNA binding (GO:0003677) and regulation of DNA-templated transcription (GO:0006355) (Fig. 12D), indicating the potential role of regulatory relationships of *KNOX* genes in Moso bamboo.

### Prediction of downstream target genes and associated KEEG pathway analysis

**Fig. 13:**
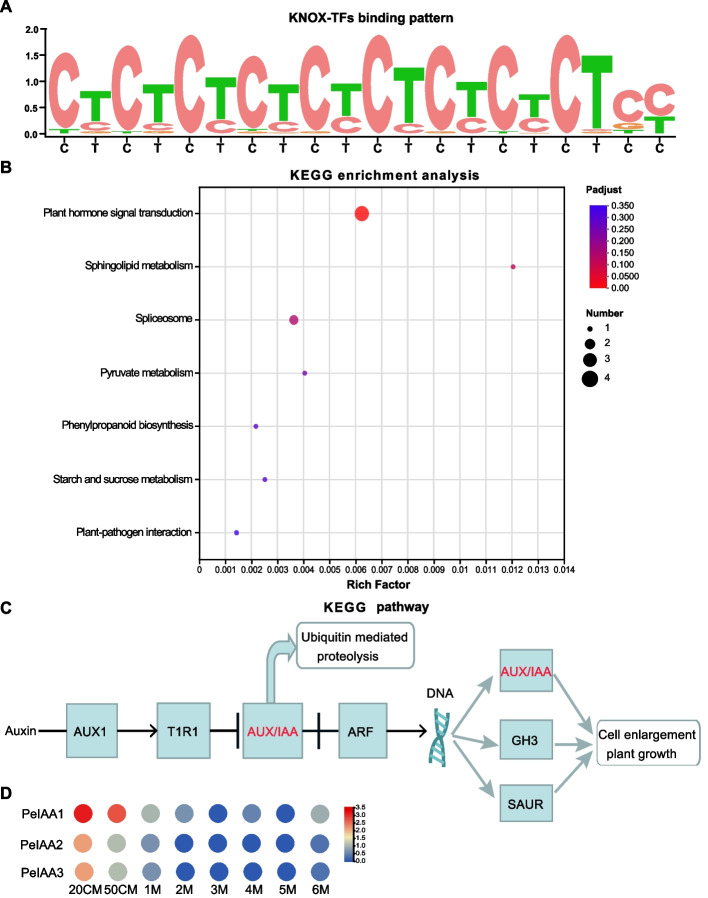
Downstream target gene binding mode and associated KEGG enrichment analysis. **A** Conserved motifs of *PeKNOX* downstream target gene binding sites. A target gene associated with this predicted binding pattern was identified. **B** KEGG enrichment analysis of *PeKNOX* target genes. Genes in the pathway are represented by points on the vertical axis, rich factors on the horizontal axis, and their sizes on the points The first 20 enrichments showed a *p*-value < 0.05. **C** IAA signal transduction pathway. **D** Heatmap for the visualization of expression analysis of PeKNOX target genes: *PeIAA1* (*PH02Gene08185*), *PeIAA2* (*PH02Gene40027*), and *PeIAA3* (*PH02Gene50429*)

Using the target prediction database, we identified 44 downstream target genes associated with the binding patterns of *PeKNOX* (Table S3). The accompanying bar plot visually represents the frequency of motif counts detected at these binding sites (Fig. 13A). KEGG functional enrichment analysis was performed for the relevant target genes and revealed that the *P*-values of the top 20 enrichment results were all less than 0.05 (Table S4). The downstream target genes are mainly enriched in plant hormone signal transduction (Fig. 13B). Among these genes, three are significantly involved in the IAA signaling pathway, implying their role in regulating cell expansion, plant growth, and development (Fig. 13C) [31]. These genes are *PeIAA1* (*PH02Gene08185*), *PeIAA2* (*PH02Gene40027*), and *PeIAA3* (*PH02Gene50429*). Furthermore, their expression profiles obtained from transcriptome data (Fig. 13D) demonstrate that the downregulation of AUX/IAA genes within the IAA signaling pathway significantly affects both cell enlargement and overall plant growth.

## Discussion

Homeobox genes, such as *KNOTTED1*-like homeobox (*KNOX*), are essential for plant growth and development and, in particular, influence the development of various plant organs through the maintenance of meristematic activity [32, 33]. However, to our knowledge, only one study has investigated the function of *KNOX* genes in Moso bamboo [27]. Several studies have reported that the mechanisms of rapid shoot growth are controlled by cell division, rapid elongation, and secondary thickening of cell walls. These growth processes are closely linked to hormones such as auxin and gibberellin and their potential downstream target genes that control the shoot development [2, 34–38]. Despite these advances, the mechanism controlling the rapid growth remains a mystery.

Therefore, the current study is dedicated to unraveling the potential roles of *KNOX* genes in Moso bamboo and identified 24 *PeKNOX* genes, named *PeKNOX01*-*PeKNOX24* based on their sequence data and chromosome location. The number of members of the *KNOX* gene family varies among species. In particular, *Arabidopsis*, Moso bamboo, and rice have 9, 24, and 13 members, respectively (Fig. 2). The *KNOX* gene family were divided into three categories: Class I, Class II, and KNATM through evolutionary analysis. The phylogenetic relationship of *PeKNOX* genes is consistent with a previous study [9].

Gene duplication, a fundamental process in molecular evolution, leads to evolutionary processes [39]. There are three main evolutionary modes of gene replication: segmental duplication, tandem duplication, and translocation events. Segmental and tandem duplications are commonly responsible for the amplification of plant gene families [40]. Although plant genomes contain numerous gene families, the size of these gene families can vary among species due to lineage-specific expansions. In the case of Moso bamboo, we observed 29 gene pairs with a collinear relationship to rice, while only 2 gene pairs were identified between Moso bamboo and *Arabidopsis*, possibly due to the evolutionary separation between monocots and dicots.

In addition, the number of *KNOX* genes in Moso bamboo was higher than that of rice, perhaps because gene duplication plays an important role in amplification of *PeKNOX* gene family. To explore this further, we performed a genome collinearity analysis of *PeKNOX* genes and found that 16 pairs of genes arose from tandem duplication, with only *PeKNOX01* and *PeKNOX12* showing no evidence of duplication. Tandem repeats can be detected in several members of the same gene family or close intergenic regions [41]. Furthermore, genome-to-genome homology analysis was performed between Moso bamboo and *Arabidopsis* and rice. The rice *KNOX* genes showed corresponding vertical homology genes with Moso bamboo, and most of them had more than two vertical homology genes. This suggests that Moso bamboo underwent additional genome-wide replication events during its evolutionary history [42]. The collinear relationship between *KNOX* members in Moso bamboo and *Arabidopsis* is consistent with the evolutionary relationship between dicots and monocots [43].

Plants contain several conserved genes, including the KNOX gene family [44]. In the current study, the analysis of *PeKNOX* gene structure revealed that both the class I and II families have four to six non-coding regions and approximately two to five introns in each gene (Fig. 1A). However, *PeKNOX13* gene has only one non-coding region compared to the other *PeKNOXs* genes*. PeKNOX* genes within the same class typically share similar biological functions. Furthermore, the motif analysis revealed similarities in the conserved structural domains of distinct gene members belonging to the same class. Nevertheless, some differences were noted between members of different subclasses. Particularly, *PeKNOX* genes in class II lack the ELK domain, whereas *PeKNOX* genes in class I contain an ELK domain (Fig. 3A). The conserved structural domains of KNOX1, KNOX2, and Homeobox_KN were detected in all *PeKNO*X genes (Fig. 3), suggesting that the structural domains of the KNOX family are highly conserved during evolution.

*Cis*-acting elements located upstream of transcriptional initiation sites are primarily responsible for regulating gene expression in the promoter region and are essential for plant development, growth, and signaling [45]. In the current study, the abundance of P-box and CAT-box *cis*-elements in Class I genes of Moso bamboo suggests their involvement in regulating growth and promoting meristem development (Fig. 1B, C). A previous study revealed that the *KNOX* gene functions as a positive regulator of stem cell maintenance in rice somatic embryogenesis through cytokinin-KNOX signaling [46]. Several Class I genes promote the growth of lateral branches and internodes.

In addition, most *cis*-regulatory elements are related to hormone response and can interact to control biological processes [22]. Plant growth and development-associated *cis*-elements, such as MYC, can be recognized by basic helix loop helix (bHLH) TFs to activate gene expression, thereby regulating plant growth and development in response to Jasmonic Acid (JA) signaling [47]. Moreover, plants have complex systems to respond to both biotic and abiotic stresses. The *KNOX* gene has been found to enable the cotton plant to respond to different stresses while also participating in plant growth and development [9]. Based on this evidence, we hypothesized that *PeKNOX* gene could influence the stress response of plants. The present study revealed several motifs were related to hormone regulation pathways in *PeKNOX* promoters. Furthermore, it revealed that Class I and Class II *PeKNOX* genes have distinct roles in regulating growth, development, and responses to stress.

TFs are essential for regulating plant growth and stress responses [48]. The present study showed that three *PeKNOX* genes could bind to the promoter region of *AUX/IAA* genes (*PeIAA1*, *PeIAA2*, and *PeIAA3*) (Fig. 13C, D) and decrease their expression during bamboo shoot development (Fig. S1). The Aux/IAA genes influence various organizational and developmental responses such as root or branch gravitropism, lateral root formation, branch tip dominance, stem elongation, leaf expansion, and leaf formation in darkness. This reduced growth hormone response underscores the potential significance of the Aux/IAA protein in regulating the growth hormone response [49]. This suggests that *PeKNOX* genes could inhibit the expression of *AUX/IAA* genes, thereby enhancing the activation of ARF TFs and promoting the growth and development of Moso bamboo shoots, particularly during the rapid growth. This underscores the critical importance of *PeKNOX* TFs in the transition of shoot development during the rapid growth. The binding mechanism between the *KNOX* gene and the *AUX/IAA* promoter element of the target genes should be confirmed and validated by a series of molecular tools in the future. The present study represents a crucial step toward unraveling the molecular mechanisms governing the rapid growth of bamboo shoots and developing a signal regulation network model incorporating *KNOX* genes.

## Conclusion

In conclusion, the genome-wide analysis of the KNOX gene family identified 24 *PeKNOX* genes in Moso bamboo and provided valuable insights into their roles in promoting the rapid shoot growth and their regulatory networks. Subcellular localization analysis revealed that PeKNOX proteins are primarily located in the nucleus, and the structural analysis unveiled highly conserved domains, such as KNOX1, KNOX2, ELK, and Homeobox_KN, emphasizing *PeKNOXs* role in transcriptional regulation and fundamental biological processes. *Cis*-acting element analysis in *PeKNOX* gene promoters revealed numerous elements associated with plant growth, development, hormone response, and stress tolerance. The phylogenetic analysis showed that *PeKNOX* genes share a closer evolutionary relationship with rice *KNOX* genes. Moreover, the expansion of PeKNOX gene family was primarily driven by tandem duplications. Furthermore, we analyzed PeKNOX gene expression in Moso bamboo using 31 distinct transcriptome datasets, revealing tissue-specific expression patterns that suggest these genes play roles in various aspects of bamboo development and growth. Several *PeKNOX* genes, particularly those in profile 7, exhibited a strong positive correlation with the shoot development, emphasizing their involvement in promoting the rapid growth. Analysis of downstream target genes and KEGG pathway highlighted *PeKNOX* gene involvements in the plant hormone signal transduction pathway. Notably, three *PeKNOX* target genes (*PeIAA1*, *PeIAA2*, and *PeIAA3*) are significantly associated with the IAA signaling pathway, suggesting their role in regulating cell expansion, plant growth, and development. This study provides valuable insights into the function of the *KNOX* gene family in the rapid growth and development of Moso bamboo.

## Materials and methods

### Plant materials and stress treatments

Tissue samples were collected from Moso bamboo shoots at various growth stages in bamboo forests in Guilin, Guangxi Province, with the landowners' consent. Moso bamboo shoots were collected at heights of 20 cm, 50 cm, 1 m, 2 m, and 3 m during the rapid growth phase. The collected tissue samples were promptly frozen in liquid nitrogen and kept at − 80 °C until RNA extraction [50]. Three biological replicates of each tissue type were used for tissue-specific expression analyses.

### Identification of KNOX gene family in Moso bamboo

Moso bamboo protein sequence was downloaded from the National Center for Gene Research (http://server.ncgr.ac.cn/bamboo/index.php). In addition, to identify the KNOX gene family, the KNOX domain hidden Markov models (HMMs) were obtained from the Pfam protein family database [51, 52]. The KNOX gene family were identified using HMMER 3.0. The E-value measures the random probability of finding a specific match in a database search, with a smaller E-value indicating higher significance and reducing the likelihood of finding highly similar sequences [53]. Genes with a search threshold of an E-value of 10^–15^ were further assessed. The ExPASy ProtParam website (https://web.expasy.org/protparam/) was used to forecast protein characteristics, including molecular weight, isoelectric point, and grand average of hydropathicity [14]. Subcellular localization of proteins belonging to the KNOX family was predicted using WoLF PSORT [54].

### Gene structure and cis-acting elements analysis

The intron–exon distributions of the *KNOX* genes were determined using the GFF annotation file from Moso bamboo genome. PlantCare (http://bioinformatics.psb.ugent.be/webtools/plantcare/html) was used to identify *cis*-acting elements in the *KNOX* genes. Furthermore, TBtools were used to visualize the results to identify the transcription start locations of each gene's *cis*-regulatory elements [55].

### Phylogenetic tree, conserved motifs, and domains analysis

Whole genome information for *Arabidopsis* and rice were downloaded from the *Arabidopsis* Information Resource, version 10 (TAIR 10) (http://www.arabidopsis.org) and the RGAP 7 database (http://rice.plantbiology.msu.edu/), respectively. The KNOX protein sequences from *Arabidopsis*, rice, and Moso bamboo were aligned using ClustalX. Subsequently, to gain insights into the phylogenetic relationships among these sequences, MEGA 7.0 was used to construct the phylogenetic tree using the neighbor-joining (NJ) method [56], with 1000 bootstrap replicates. Motifs of the KNOX proteins were then analyzed using MEME [57]. Furthermore, conserved domains of the KNOX proteins were predicted by the NCBI Batch Web CDD-Search Tool (https://www.ncbi.nlm.nih.gov/Structure/bwrpsb/bwrpsb.cgi). Finally, the protein structures were visualized using TBtools [58].

### Chromosomal distribution and gene duplication analysis

Chromosomal length and the *KNOX* gene location were determined using data obtained from the Phytozome database. MCScanX46 was used to identify duplication events and co-linear interactions among the KNOX protein sequences in *Arabidopsis* and rice. TBtools software was used to generate Super Circos [59].

### Expression analysis of KNOX genes in Moso bamboo based on the RNA-Seq data

The *KNOX* expression profile was comprehensively analyzed from 31 distinct transcriptome datasets of Moso bamboo downloaded from the NCBI sequences, focusing on spatiotemporal tissue expression profiles in the following key datasets: (i) the samples from the roots, stems, panicles, and leaves (PRJEB2956), (ii) the samples from shoot tissue obtained at different heights of 20 cm, 50 cm, 1 cm, 2 cm, 3 cm, 4 cm, 5 cm, and 6 cm (PRJNA414226), and (iii) the seedling root tissues treated with 5 mM gibberellic acid (GA) or 5 mM naphthaleneacetic acid (NAA) (PRJNA413166). Heatmaps of expression levels were constructed using log10-transformed transcripts per million transcripts (TPM) values [60, 61].

### GO enrichment analysis and protein interaction networks

The KNOX protein functions were categorized, into biological processes, cellular components, and molecular functions, using the GO database (Gene Ontology; http://www.geneontology.org/). Fisher's exact test was used to compare the enrichment of PeKNOX with the entire GO database. The *P*-value (*P* adjust) of functions that were substantially enriched under the Bonferroni correction (Bonferroni52) was less than 0.05 [62]. The STRING database (https://STRING-db.org) was used to compare and associate nodes among the KNOX protein sequences [63]. Cytoscape (V3.7.1) was used to visualize the resulting network [64].

### Time series expression analysis and qRT-PCR assays

Time-series analysis was used to analyze the *KNOX* gene expression patterns over a period of time based on STEM. In addition, functional class enrichment was used to identify the biological roles of *KNOX* genes. Temporal clustering based on STEM was used to identify gene expression during the rapid growth of bamboo shoots at five different time points, with a significant trend p-value set at 0.05 [65]. For qRT-PCR analysis, Moso bamboo samples, stored at -80 °C, were treated with FastPure Plant Complete RNA Isolation Package into fine powder to extract total RNA. Subsequently, cDNA was synthesized using HiScript@ III 1st Strand cDNA Synthesis Kit, following manufacturer’s instructions. Quantification of threshold cycle (CT) value was achieved using the 2-^ΔΔCT^ method. Moreover, ANOVA was used to assess treatment differences based on standard deviation (SD) [66].

### Subcellular localization

The subcellular localization of *PeKNOX08* and *PeKNOX23* genes was determined in *Nicotiana benthamiana* (tobacco) cells using primers designed with similar coding sequences by homologous recombination. The coding sequences (CDS) of *PeKNOX08* and *PeKNOX23* genes without stop codons were amplified and cloned into pCAMBIA1300-GFP vectors. The empty pCAMBIA1300 CaMV 35S:GFP vectors were used as controls. Positive clones were confirmed by PCR and DNA sequencing, and the plasmid constructs were transformed into *Agrobacterium tumefaciens* strain GV3101 using a conventional freeze–thaw method. Tobacco plants were grown in a greenhouse at a temperature of 23 °C for about one month. The recombinant vector was injected into the epidermis of the third or fourth leaf of tobacco plant. The expression of fusion proteins was analyzed using laser confocal microscopy [67].

### Construction of KNOX gene regulatory networks and GO enrichment analysis

The PlantPAN database was used to search for potential transcription factor binding motifs located 1000 bp upstream of each gene. The Binding Motif was used to search for the presence of Binding Sites in the promoter regions of the *KNOX* genes, and the candidates were screened based on a correlation coefficient greater than 0.80. To further analyze the motifs located within the promoter regions and identify downstream target genes regulated by the *KNOX* genes, JASPAR (http://jaspar.genereg.net/) and FIMO (http://meme-suite.org/tools/fimo) web tools were used [68]. The candidate motifs were screened based on a correlation coefficient greater than 0.95 and a P-value ≤ 1 × e^−8^. Similarly, these criteria were carried out to identify target genes associated with the motifs. Based on the screening results, gene regulatory networks were constructed using Cytoscape software. The upstream transcription factors and downstream target genes of the *KNOX* genes were analysed for GO enrichment analysis using GO-tools.

### Prediction of target genes and KEGG pathway enrichment analysis

As described above in Sect. 3.10, downstream target genes were identified using JASPAR and FIMO web tools. Then, the WebLogo program (http://weblogo.threeplusone.com/) was used to generate the conserved motif logos. In addition, functions of the *KNOX* target genes were annotated from the Kyoto Encyclopedia of Genes and Genomes (KEGG) databases. Major-BioCloud (https://cloud.majorbio.com) was used for the analysis and visualization. The overall expression levels of downstream target genes in bamboo shoots at different heights from 20 cm to 6 m were determined via transcript analysis using the fragments per kilobases per million (FPKM) mapped reads method. Analysis of gene network pathway was performed on genes that represented significantly regulated pathways [62].

### Supplementary Information

**Supplementary Material 1.**

**Supplementary Material 2.**

**Supplementary Material 3.**

**Supplementary Material 4.**

**Supplementary Material 5.**

## Data Availability

This published article and its supplementary information files contain all the data generated or analyzed during this investigation.

## Abbreviations

MCScanX: Multiple Collinearity Scan toolkit
STEM: Short Time-series Expression Miner
KEGG: Kyoto Encyclopedia of Genes and Genomes databases
