# Supplementary material for: Genome-wide analysis of the KNOX gene family in Moso bamboo: insights into their role in promoting the rapid shoot growth
Source: BMC Plant Biol. 2024 Mar 25;24:213. doi: 10.1186/s12870-024-04883-2 (PMC10962149; doi:10.1186/s12870-024-04883-2)
Supplement: Supplementary file 1 — Supplementary Material 1. [file 12870_2024_4883_MOESM1_ESM.pdf]

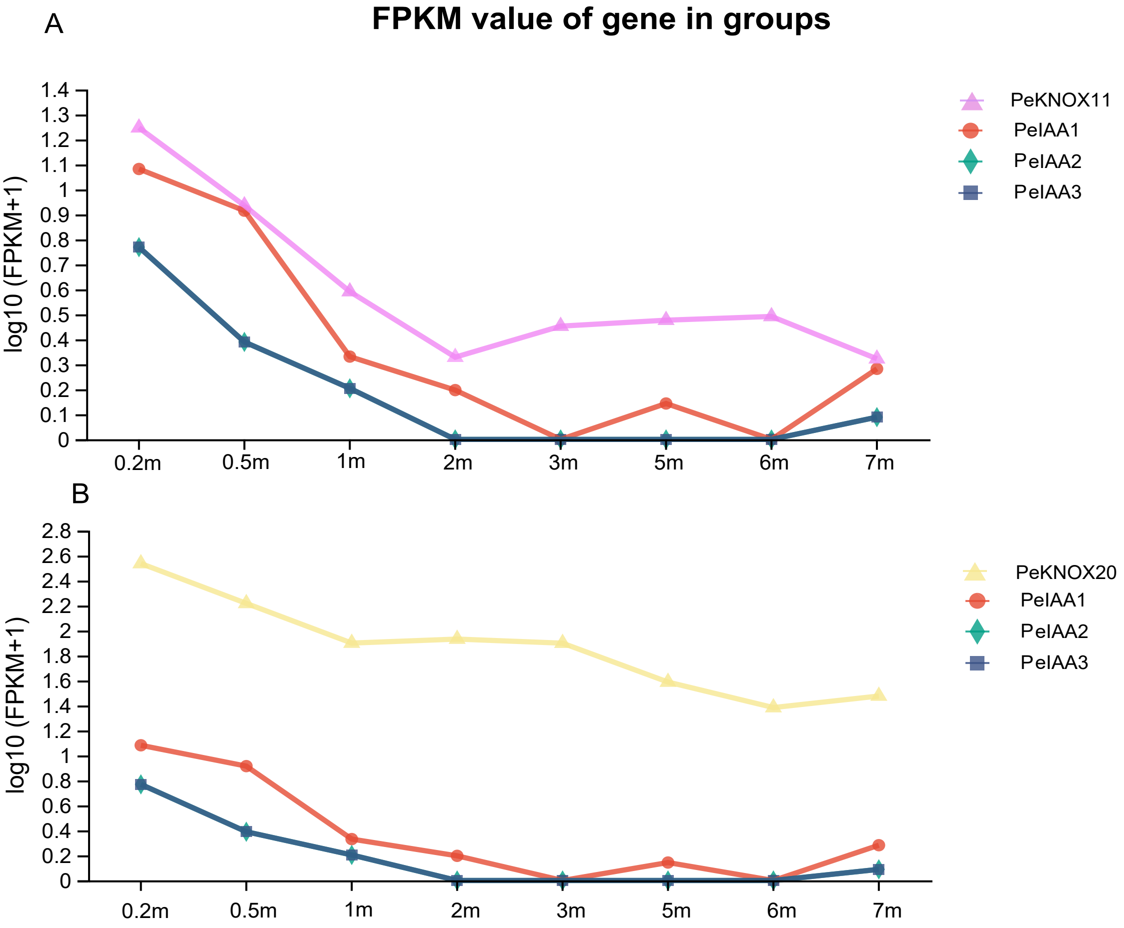

Fig. S1. The expression levels of major transcriptional regulators and downstream target genes were measured at various growth and development stages.
